# EMBL-MCF 2.0: an LC-MS/MS method and corresponding library for high-confidence targeted and untargeted metabolomics using low-adsorption HILIC chromatography

**DOI:** 10.1007/s11306-024-02176-1

**Published:** 2024-10-13

**Authors:** Svitlana Dekina, Theodore Alexandrov, Bernhard Drotleff

**Affiliations:** 1 Metabolomics Core Facility, European Molecular Biology Laboratory (EMBL), Heidelberg, Germany; 2 Structural and Computational Biology Unit, EMBL, Heidelberg, Germany; 3 Molecular Medicine Partnership Unit, EMBL and Heidelberg University, Heidelberg, Germany; 4 Bio Studio, BioInnovation Institute, Copenhagen, Denmark; 5 Department of Pharmacology, University of California San Diego, La Jolla, CA, USA; 6 Department of Bioengineering, University of California San Diego, La Jolla, CA, USA; 7 Department of Biomedicine, O.V. Bogatsky Physico-Chemical Institute of the National Academy of Sciences of Ukraine, Odesa, Ukraine

**Keywords:** LC-MS/MS, Spectral library, Untargeted metabolomics, Targeted metabolomics, Low-adsorption chromatography

## Abstract

**Introduction:**

Over the past two decades, liquid chromatography-mass spectrometry (LC-MS)-based metabolomics has experienced significant growth, playing a crucial role in various scientific disciplines. However, despite these advance-ments, metabolite identification (MetID) remains a significant challenge. To address this, stringent MetID requirements were established, emphasizing the necessity of aligning experimental data with authentic reference standards using multiple criteria. Establishing dependable methods and corresponding libraries is crucial for instilling confidence in MetID and driving further progress in metabolomics.

**Objective:**

The EMBL-MCF 2.0 LC-MS/MS method and public library was designed to facilitate both targeted and untargeted metabolomics with exclusive focus on endogenous, polar metabolites, which are known to be challenging to analyze due to their hydrophilic nature. By accompanying spectral data with robust retention times obtained from authentic standards and low-adsorption chromatography, high confidence MetID is achieved and accessible to the metabolomics community.

**Methods:**

The library is built on hydrophilic interaction liquid chromatography (HILIC) and state-of-the-art low adsorption LC hardware. Both high-resolution tandem mass spectra and manually optimized multiple reaction monitoring (MRM) transitions were acquired on an Orbitrap Exploris 240 and a QTRAP 6500+, respectively.

**Results:**

Implementation of biocompatible HILIC has facilitated the separation of isomeric metabolites with significant enhancements in both selectivity and sensitivity. The resulting library comprises a diverse collection of more than 250 biologically relevant metabolites. The methodology was successfully applied to investigate a variety of biological matrices, with exemplary findings showcased using murine plasma samples.

**Conclusions:**

Our work has resulted in the development of the EMBL-MCF 2.0 library, a powerful resource for sensitive metabolomics analyses and high-confidence MetID. The library is freely accessible and available in the universal .*msp* file format under the CC-BY 4.0 license: mona.fiehnlab.ucdavis.edu https://mona.fiehnlab.ucdavis.edu/spectra/browse?query=exists(tags.text:%27EMBL-MCF_2.0_HRMS_Library%27), EMBL-MCF 2.0 HRMS https://www.embl.org/groups/metabolomics/instrumentation-and-software/#MCF-library.

**Supplementary Information:**

The online version contains supplementary material available at 10.1007/s11306-024-02176-1.

## Introduction

Driven by continuous developments in instrumentation, protocols and software, liquid chromatography-mass spectrometry (LC-MS)-based profiling of small, polar molecules in biological systems, here referred to as metabolomics, has matured to become a widely adopted technique across various scientific domains. Despite the significant progress made over the past two decades, the metabolomics community still faces persistent challenges, in particular metabolite identification (MetID) (Cui et al., 2018).

Undoubtedly, accurate MetID is crucial for the field to maintain its relevance and make substantial contributions to the interpretation of biological processes. Consequently, stringent criteria have been established, imposing demands on researchers to match experimental data to at least two orthogonal characteristics of authentic reference standards. These rigorous MetID requirements, coupled with the proposition of distinct confidence levels, have been communicated in a series of standardized reporting guidelines introduced by the Metabolomics Standard Initiative (Sumner et al., 2007; Fiehn et al., 2007) and have also been endorsed by numerous experts in the field (Schymanski et al., 2014; Schrimpe-Rutledge et al., 2016; Creek et al., 2014). Unfortunately, the clear reporting standards, designed to ensure the reliability of results, are frequently disregarded. In fact, recent meta-analyses revealed that only a minority of published works (~ 20%) adhere to MetID confidence level 1 (Kodra et al., 2022), which ideally involves matching of acquired retention time, accurate mass, isotope pattern, and MS/MS fragmentation. This finding underscores the paramount importance of high-quality spectral libraries in advancing metabolomics research (Bittremieux et al., 2022). While several databases such as GNPS, MoNA, METLIN MS2, METLIN-MRM, mzCloud, and HMDB offer valuable information, they are not without limitations. These limitations include factors like commercial availability, the presence of in silico and non-experimental spectra, low mass accuracy, limited mass resolution, platform-specific constraints, and the absence of (relative) retention time information with corresponding LC-MS workflows.

Due to the vast complexity of the metabolome, the presence of isomers, isobars, in-source fragments, and adducts may lead to false interpretation of spectral data, specifically for non-expert users enabled by the broader accessibility and lowered entry barriers into LC-MS applications. In a separate study (Theodoridis et al., 2023), evident misannotations in various published metabolomics studies have been identified, underscoring the growing apprehension regarding the validity of many reported metabolite annotations. This concerning trend not only jeopardizes the credibility of metabolomics, but also risks erroneous conclusions and inefficient resource allocation, hindering overall scientific progress. Simple and appropriate tools for high confidence MetID therefore have to be offered to the growing community.

To address issues related to selectivity, sensitivity, and metabolite coverage in LC-MS metabolomics, continuous optimization of LC conditions must be considered. The introduction and further development of hydrophilic interaction liquid chromatography (HILIC) has significantly expanded the analytical toolkit, offering improved retention capabilities for polar and hydrophilic molecules (Gilar et al., 2022; Fecke et al., 2023; Lioupi et al., 2022; Serafimov et al., 2022). However, researchers are confronted with enhanced complexity compared to reversed-phase (RP) LC. HILIC experiences extended equilibration times, challenges in predicting and modeling retention mechanisms, and reduced repeatability due to increased sensitivity to variations in mobile phase composition, column conditions and sample content.^14^ Moreover, the presence of common functional groups such as phosphates and carboxylates add further complexity to metabolomics analysis due to their non-specific adsorption onto metal and stainless-steel surfaces within LC-MS instrumentation. To mitigate issues such as peak tailing and the potential loss of metabolites containing these chemical moieties, the development and commercial availability of biocompatible materials for analytical instrumentation and column hardware have become essential. These low-adsorption materials, like PEEK, titanium, MP35N (a nickel-cobalt alloy), and hybrid surface technology (HST) exhibit enhanced separation efficiency and yield higher sensitivity and recovery rates (DeLano et al., 2021; Walter et al., 2022).

The primary objective of this work is to introduce an open-source LC-MS/MS library and a corresponding analytical method for targeted and untargeted metabolomics applications. Our approach leverages the latest advancements in HILIC separations and utilizes low-adsorption hardware to address common repeatability challenges and effectively separate prominent isomeric compounds. Furthermore, we offer a carefully curated library of high-resolution MS/MS data, which enhances MetID accuracy in untargeted analysis. Additionally, for the metabolites included in the library, we provide optimized and selective multiple reaction monitoring (MRM) transitions, thus enabling running matching or complimentary sensitive and validated detection of those metabolites in a targeted fashion.

The EMBL-MCF 2.0 library is the next version of the previous EMBL-MCF library (Phapale et al., 2021). This version employs robust low-adsorption chromatography and provides retention time information in universal .*msp* format as well as MRM information for targeted applications. Metabolite features of the library were selected to cover solely polar molecules of biological relevance that show sufficient retention and yield quantifiable peaks. Moreover, a tool for automatic noise reduction was developed and applied to remove low-abundant MS/MS fragments from the high-resolution spectral data and corresponding HMDB IDs were added directly to .*msp* files, streamlining subsequent data processing efforts such as pathway enrichment. The *EMBL-MCF 2.0* library is available for download at mona.fiehnlab.ucdavis.edu https://mona.fiehnlab.ucdavis.edu/spectra/browse?query=exists(tags.text:%27EMBL-MCF_2.0_HRMS_Library%27) and EMBL-MCF 2.0 HRMS https://www.embl.org/groups/metabolomics/instrumentation-and-software/#MCF-library, and will be iteratively extended and updated after successful implementation and validation of additional metabolites.

## Materials and methods

### Materials

High purity formic acid, ammonium hydroxide, ammonium acetate, and ammonium formate were purchased from Sigma-Aldrich (Germany). Acetonitrile, methanol and water of Ultra LC-MS grade (Chemsolute, Germany) were used as solvents for mobile phases and preparation of stock solutions. Reference standards were purchased from MetaSci Inc. (ON, Canada) and Sigma-Aldrich.

### Preparation of reference standards mixes

Stock solutions of the individual analytes were prepared at concentrations of 1.0 mg/mL in 80% methanol. To reduce the number of required injections, reference standard mixes consisting of 10 individual compounds were prepared at a uniform concentration of 10 µg/mL in 80% methanol. The analyte content of mixtures was chosen in a way to avoid interference from isomers and potential isomers resulting from common adducts and in-source fragmentation. Final mixtures were used to acquire high-resolution spectra for untargeted analysis and for compound tuning and optimization for targeted analysis. In addition, reference standard mixes were also combined (50:50 dilution) with methanolic extracts of yeast and mammalian cells to check for selectivity in true biological matrix. Individual stocks and reference mixes were stored at -20 °C.

### Preparation of plasma samples

Plasma samples (100 µL) were extracted by adding 400 µL of 80% methanol. After 20 min of incubation at -20 °C, the protein-precipitated extracts were centrifuged at 14 000 × g for 15 min at 4 °C and supernatants were carefully transferred to new tubes without disturbing the pellet. Quality control (QC) samples were prepared by pooling 50 µL aliquots of each processed sample (Kirwan et al., 2022). Control blanks were prepared in the same way as samples, using Ultra LC-MS grade water instead of plasma. Injection volume was set to 3 µL for all samples and platforms.

### Liquid chromatography-mass spectrometry

LC-MS/MS high-resolution untargeted analysis was carried out on a biocompatible Vanquish Horizon UHPLC system (MP35N-based) coupled to an Orbitrap Exploris 240 mass spectrometer (Thermo Scientific, MA, USA). Targeted LC-MS/MS analysis was performed on an Exion LC AD system hyphenated to a QTRAP 6500+ (Sciex, MA, USA).

### Liquid chromatography

Chromatographic separation was carried out on a sulfobetaine-based Atlantis Premier BEH Z-HILIC column (Waters, MA, USA; 2.1 mm x 100 mm, 1.7 μm) at a flow rate of 0.25 mL/min. The mobile phase consisted of water: acetonitrile (9:1, v/v; mobile phase A) and acetonitrile: water (9:1, v/v; mobile phase B), which were modified with a total buffer concentration of 10 mM ammonium acetate (negative mode) and 10 mM ammonium formate (positive mode), respectively. The aqueous portion of each mobile phase was pH-adjusted (negative mode: pH 9.0 via addition of ammonium hydroxide; positive mode: pH 3.0 via addition of formic acid). In order to ensure best possible repeatability of chromatographic retention times, preparation of mobile phases was conducted as precisely as possible, using volumetric flasks and adhering to a detailed standard operating procedure. The following gradient was applied: 0–2 min: holding 95% mobile phase B; 2–14.5 min: ramping to 60% mobile phase B; 14.5–16 min: holding 60% mobile phase B; 16–16.5 min: increasing to 95% mobile phase B; 16.5–20 min: holding and re-equilibration at 95% mobile phase B. Column temperature was maintained at 40 °C, the autosampler temperature was set to 4 °C and sample injection volume was 3 µL. Further details about retention time are listed in Supplementary Tables S1 and S2.

### Untargeted metabolomics: Orbitrap Exploris 240

Analysis was performed in positive and negative H-ESI (heated-electrospray ionization) mode. For a broad coverage of metabolite features, analytes were recorded via a full scan with a mass resolving power of 120,000 over a mass range from 60 to 900 *m/z* (RF lens: 70%). To obtain MS/MS fragment spectra, data-dependent acquisition was carried out (resolving power: 15,000; scan time: 22 ms; stepped collision energies [%]: 30/50/70; cycle time: 900 ms). Ion source parameters were set to the following values: spray voltage: 3500 V / -3000 V, sheath gas: 30, auxiliary gas: 5, sweep gas: 0, ion transfer tube temperature: 350 °C, vaporizer temperature: 300 °C.

### Targeted metabolomics: QTRAP 6500+

Analysis was performed in positive and negative ionization mode using an IonDrive Turbo V electrospray ionization (ESI) source. Ion source parameters were: curtain gas (N_2_): 40 psi; nebulizer gas (zero air): 80 psi; heater gas (zero air): 40 psi; ion spray voltage: 4500 V in positive and − 4500 V in negative ionization mode; entrance potential: 10 V in positive and − 10 V in negative ionization mode; medium collision gas (N_2_) pressure; ion source temperature: 350 °C. Dwell time per MRM experiment was set to 2 ms and total cycle time (tCyc) was delimited to 900 ms in order to provide at least 10–12 data points per peak for accurate and precise peak integration and quantification. Further details about MRM parameters are listed in Supplementary Tables S3 and S4.

### Data analysis

#### High-resolution library generation

**Fig. 1:**
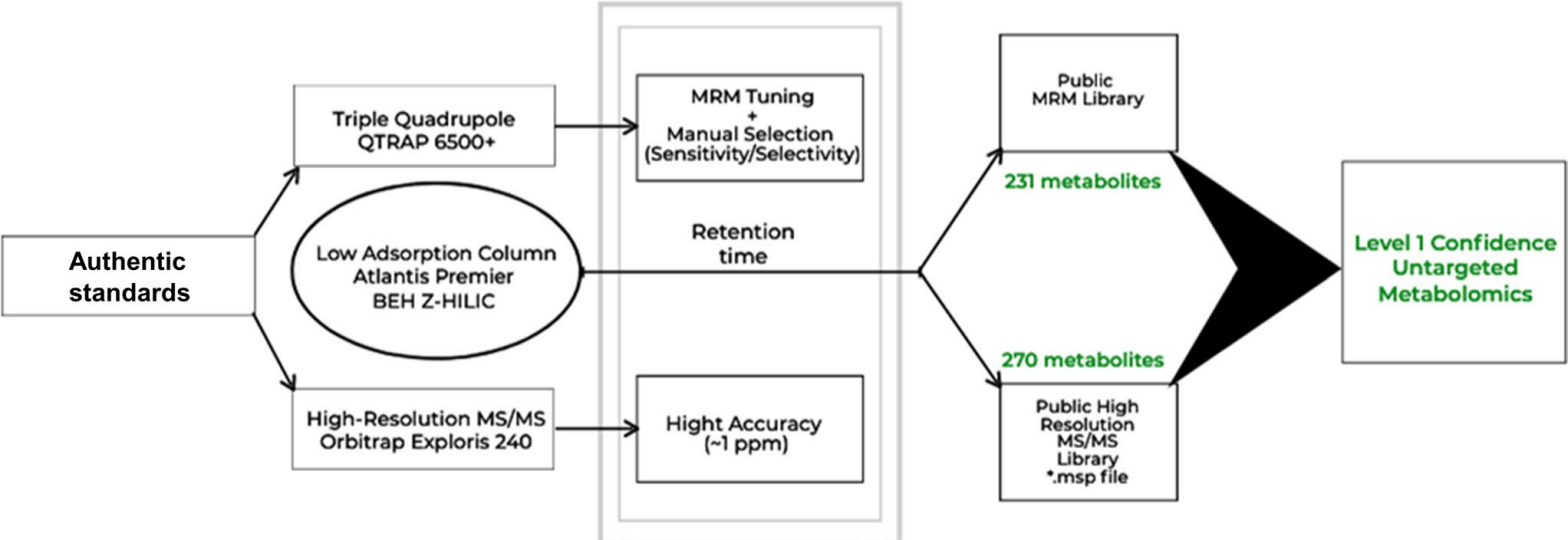
Workflow for the curation of the EMBL-MCF 2.0 LC-MS/MS spectral library

Raw data obtained from neat reference standard mixtures as well as spiked extracted biological samples were thoroughly investigated to identify prevalent adduct types. Alternative adducts with a significant portion (> 10%) of the main adduct type of respective compounds were included as separate entries in the library. To yield fragmentation-rich spectra for a diverse range of molecules, data were collected using a collision energy (CE) spread (stepped CE [%]: 30, 50, 70). Following the comprehensive curation of the data and exportation of .*msp* files, low-abundant MS/MS signals close to noise level (< 1%) were removed using a customized *R* script with a settable intensity threshold and absolute intensity values of fragments were converted to relative intensities for improved readability. Raw files of acquired mixes of authentic standards were imported to mzVault and library data was exported as .*msp* files (positive and negative mode, respectively) after careful curation. The workflow for the design and creation of the library is shown in Fig. 1.

#### MRM library generation

In contrast to untargeted high-resolution metabolomics, targeted analysis typically requires the optimization of ionization and fragmentation parameters to achieve favorable sensitivity and selectivity. To generate multiple reaction monitoring (MRM) data for the selected metabolite panel, reference standard mixes were manually tuned via direct infusion. Optimum values for declustering potential (DP), collision energy (CE), and collision cell exit potential (CXP) were assigned for the six most intense MS/MS fragments per compound. Subsequently, results from targeted LC-MS/MS analysis of standard reference mixtures and spiked biological samples were utilized to identify the two most sensitive and selective MRM transitions for inclusion in the final MRM library (Fig. 1). By providing two MRM transitions per analyte (qualifier and quantifier) whenever possible, validation of selectivity can be assured by monitoring qualifier/quantifier ratios (Domingo-Almenara et al., 2018). The final MRM parameters can be seen in Tables S3 & S4.

#### Data processing for metabolomic analysis of plasma samples

Data processing for untargeted analysis was performed using MS-DIAL (4.9.221218) (Tsugawa et al., 2015). Details on used parameter settings in MS-DIAL are described in the Supplementary Information. Targeted data acquired on the triple-quadrupole system was processed using SCIEX OS software and the default integration parameters.

## Results and discussion

**Fig. 2:**
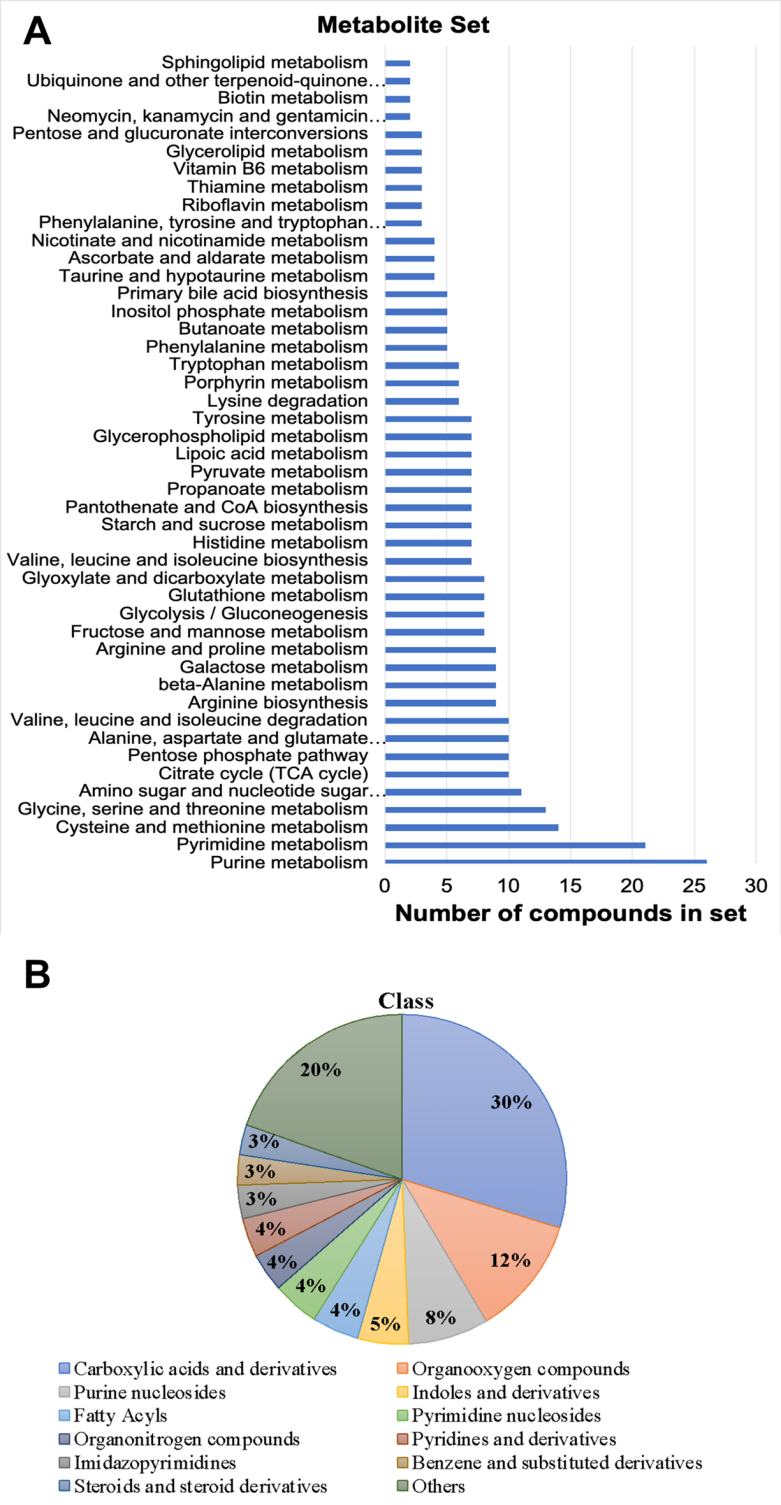
EMBL-MCF 2.0 spectral library coverage with different KEGG pathways (A) and chemical classes (B) highlighted using ClassyFire

The selection of compounds included in the library prioritized biological relevance, with a primary focus on endogenous mammalian metabolites. Notably, the library does not encompass xenobiotics and drugs. Also, lipids and lipid-like molecules were omitted due to their inadequate retention and separation under the chromatographic conditions employed. However, the chosen HILIC conditions allow for the accurate analysis of additional molecules depending on their polarity. To overcome these limitations, public data for other molecular targets can be merged with the provided *.msp* files or supplemented with information from other MRM libraries, enhancing the applicability of the library for broader studies. In total, we incorporated 270 metabolites, spanning diverse metabolite classes and offering a wide representation of the known metabolome (see Fig. 2A, B).

### Chromatographic performance

**Fig. 3:**
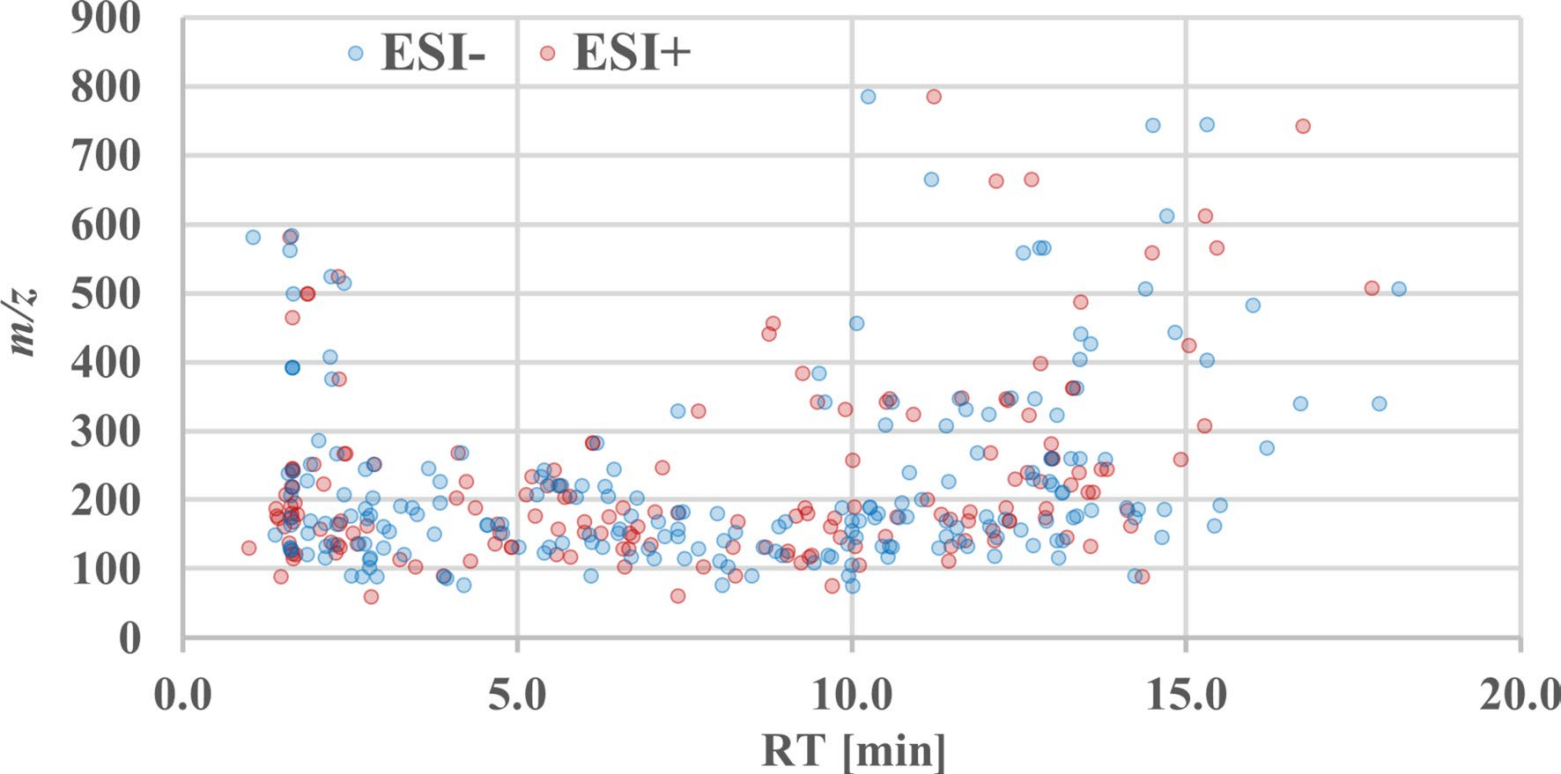
BEH Z-HILIC metabolite distribution in positive (ESI+) and negative (ESI-) ionization modes

In addition to the performance of HILIC columns in terms of retention of polar molecules, the employed Atlantis Premier BEH Z-HILIC column is constructed with low-adsorption hardware within the relevant flow path. This design significantly minimizes unwanted analyte-surface interactions, a common issue that can lead to poor peak shapes and reduced signal intensity (Tobolkina et al., 2022). In combination with a biocompatible chromatographic system, excellent peak shape with minimum tailing and separation can be achieved for multiply phosphorylated compounds. During the initial acquisition of reference standard mixtures, rigorous criteria for assessing peak width (< 0.5 min), tailing factor, and peak asymmetry were applied to select suitable metabolites and ensure the reliability and quality of chromatographic performance. In the design of the mobile phase gradient, our primary objective was to maximize the available separation space to accommodate the diverse polarity range of analytes while simultaneously ensuring efficient re-equilibration. The achieved distribution of retention times for included metabolites across the chromatographic run time is highlighted in Fig. 3.

By adhering to a detailed mobile phase preparation protocol, which involved fresh preparation of small batches (maximum batch volume of 2 L), precise pH meter-guided pH-adjustment in the aqueous portion, and the use of volumetric flasks, a retention time repeatability of < 1% CV for amino acids throughout 7 individual batches of mobile phase preparation was achieved (see Supplementary Fig. S1 and Table S5). The precise retention behavior plays a crucial role in the applicability of a HILIC-based level 1 LC-MS/MS library. Furthermore, in terms of stability, we did not observe noticeable decrease or change in performance even after conducting more than 4000 injections over several months.

**Fig. 4:**
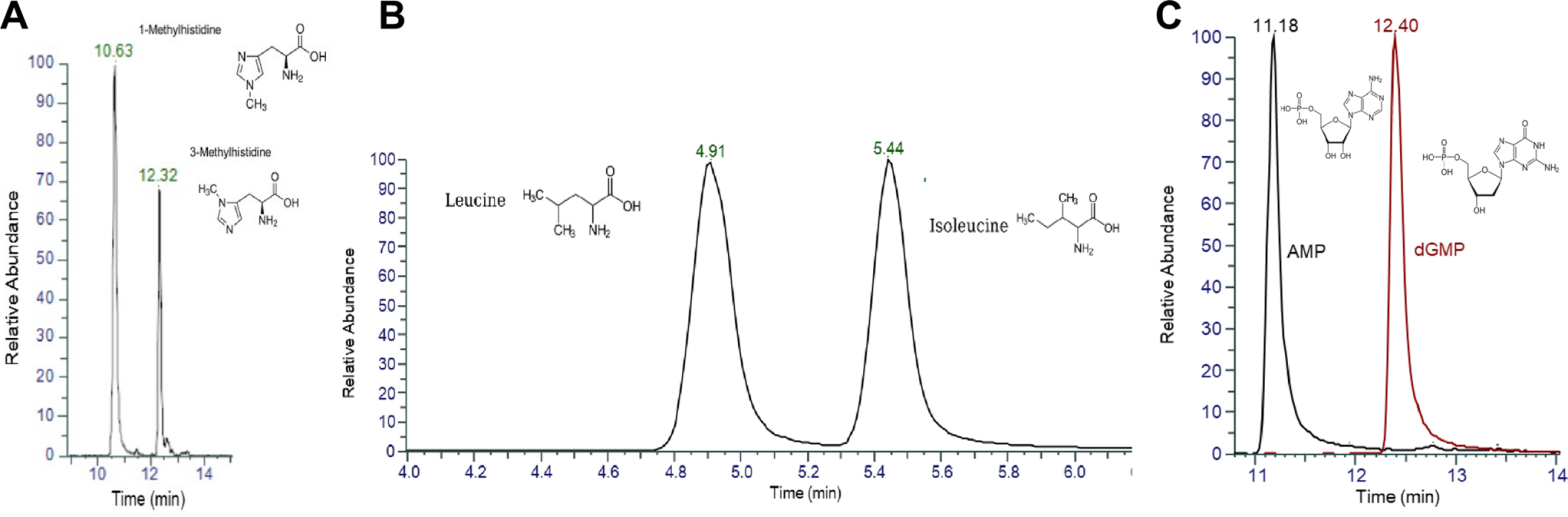
BEH Z-HILIC chromatograms of authentic standards of isomeric 1-methylhistidine and 3-methylhistidine (A) as well as leucine and isoleucine (B), AMP and dGMP (C) in ESI+

The developed method also successfully addresses challenges related to isomer selectivity. A list of chromatographically separated isomers is presented in Table S6. Exemplary separation of isomeric analyte pairs can be highlighted for: 1-methylhistidine and 3-methylhistidine (Rs_FWHM_ = 6.76); leucine and isoleucine (Rs_FWHM_ = 2.18); AMP and dGMP (Rs_FWHM_ = 3.81) (Fig. 4A-C).

The chromatographic conditions employed in this study effectively separate such analytes as NAD^+^ and NADH, NADP^+^ and NADPH from one another to prevent potential interference caused by oxidation during the ESI process (Fig. S2).

### Metabolomic analysis of plasma samples

**Fig. 5:**
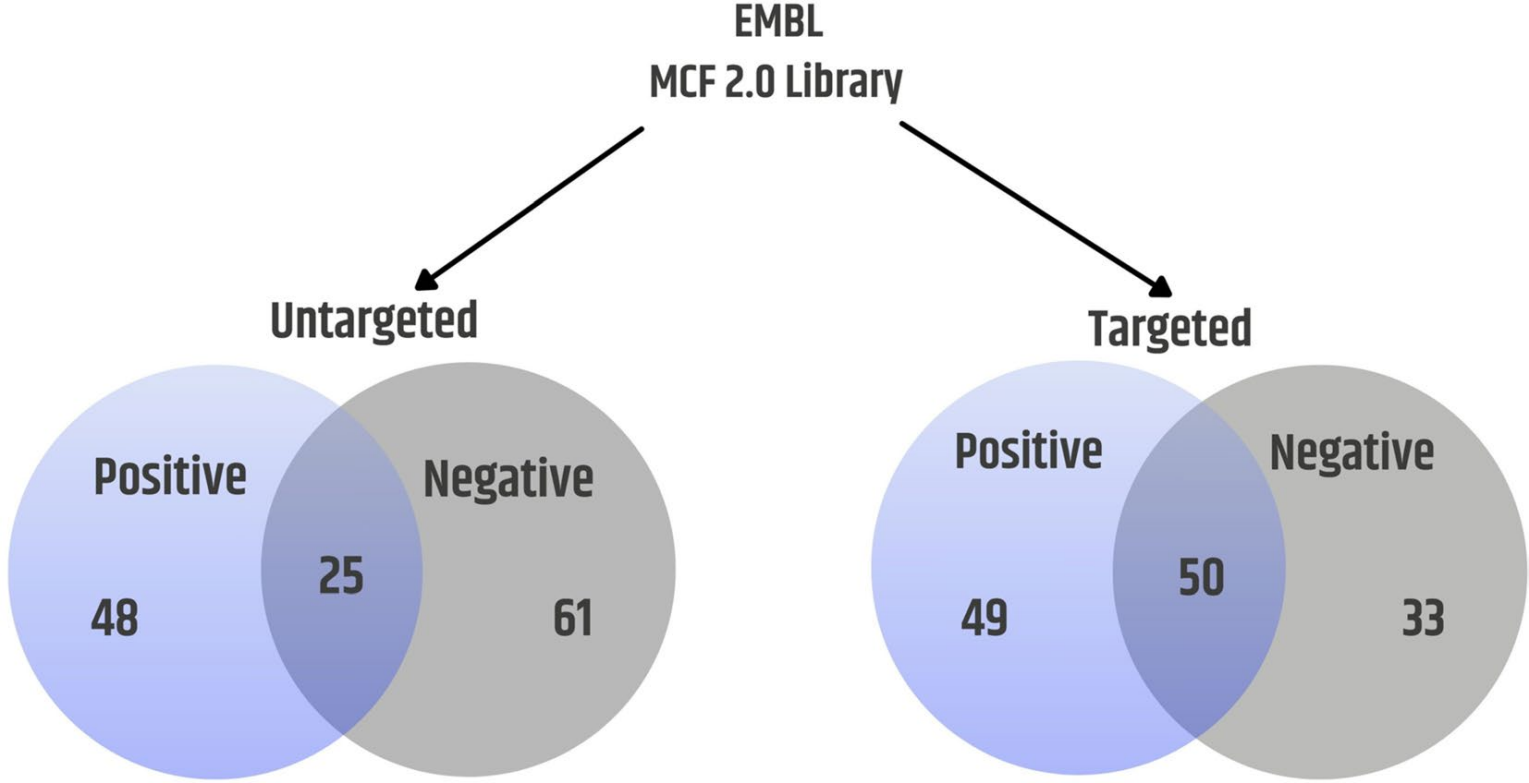
Number of detected metabolites in plasma cells with level 1 confidence

The method has been applied to investigate various biological matrices, e.g. cells, spent medium, yeast, bacteria, tissue, cerebrospinal fluid, urine and other biofluids. To demonstrate the utility of our approach, the targeted and untargeted LC-MS/MS workflow was employed for metabolomics analysis of murine plasma samples. In this example, we have confidently identified 125 distinct compounds (93 in negative, 80 in positive mode) in plasma. Average differences in observed retention times between library data and plasma samples were 5.44 ± 7.12 s in positive mode and 3.91 ± 5.30 s in negative mode. Multiple metabolites are accessible for detection in both ionization modes (Fig. 5).

For further evaluation and to determine the favorable mode of analysis for these analytes, we have calculated ratios for peak area and peak height between positive and negative mode results and compared CVs of the used quality control pool samples between modes (Table S7).

### Limitations of the library

The library for metabolite identification exhibits several limitations that should be considered. First, some challenges for isomer separation, e.g. sugar-phosphates, remain and are to be tackled by dedicated chromatographic method development. Also, for certain carboxylated compounds such as citric acid and malic acid, satisfying chromatographic results could not be achieved due to poor peak shape at given LC parameters. Another issue that applies to mass spectral libraries in general, is the limited transferability of the spectra and retention times to other laboratories and instruments, necessitating a higher retention time tolerance or adjustment of instrument fragmentation parameters. One potential improvement is the injection of a few selected standards across the entire retention time range, which allows for the conversion of retention times into relative retention time values and extrapolation of results. Nonetheless, the elution order, especially under the same liquid chromatography conditions, remains valuable information. For optimum reproducibility of retention times and method transfer between chromatographic systems, we have provided information about gradient delay volume and extracolumn volume of the used biocompatible LC in the Supplementary Information. To further enhance the utility of the provided library, it is recommended to use the provided .*msp*-file for achieving high-confidence level 1 MetID and combining it with data from public libraries for extended level 2 annotations in discovery metabolomics research.

## Conclusions

Our work represents a powerful LC-MS/MS method and library using reliable and state-of-the-art low-adsorption HILIC and curated high-quality Orbitrap mass spectra. The EMBL-MCF 2.0 library provides a straightforward tool suitable for both seasoned experts and newcomers in the field, enabling the generation of high-confidence metabolomics results. By harnessing the power of hydrophilic interaction liquid chromatography (HILIC) and low-adsorption hardware, our approach offers high selectivity and sensitivity. EMBL-MCF 2.0, containing 270 biologically relevant metabolites, spans a wide range of metabolite classes, making it a reliable resource for investigating mammalian metabolism. The library is freely accessible to the scientific community, representing an important advancement towards reliable and broad range metabolite identification, facilitating accurate and rapid generation of results to improve scientific progress in the metabolomics field.

## Electronic supplementary material

Below is the link to the electronic supplementary material.

Supplementary Material 1

Supplementary Material 2

## Data Availability

No datasets were generated or analysed during the current study.

## References

[CR1] Bittremieux, W., Wang, M., & Dorrestein, P. C. (2022). The critical role that spectral libraries play in capturing the metabolomics community knowledge. *Metabolomics: Official Journal of the Metabolomic Society*, *18*, 94. 36409434 10.1007/s11306-022-01947-y PMC10284100

[CR2] Creek, D. J., Dunn, W. B., Fiehn, O., Griffin, J. L., Hall, R. D., Lei, Z., Mistrik, R., Neumann, S., Schymanski, E. L., Trengove, R., & Wolfender, J. (2014). Metabolite identification: are you sure? And how do your peers gauge your confidence? *Metabolomics*, 10, 350–353.

[CR3] Cui, L., Lu, H., & Lee, Y. H. (2018). Challenges and emergent solutions for LC-MS/MS based untargeted metabolomics in diseases. *Mass Spectrometry Reviews*, *37*, 772–792. 29486047 10.1002/mas.21562

[CR4] DeLano, M., Walter, T. H., Lauber, M. A., Gilar, M., Jung, M. C., Nguyen, J. M., Patel, M., Bates-Harrison, A., & Wyndham, K. D. (2021). Using Hybrid Organic–Inorganic Surface Technology to mitigate analyte interactions with metal surfaces in UHPLC. *Analytical Chemistry*, *93*, 14, 5773–5781. 33798331 10.1021/acs.analchem.0c05203

[CR5] Domingo-Almenara, X., Montenegro-Burke, J. R., Ivanisevic, J., et al. (2018). XCMS-MRM and METLIN-MRM: A cloud library and public resource for targeted analysis of small molecules. *Nature Methods*, *15*, 681–684. 30150755 10.1038/s41592-018-0110-3 PMC6629029

[CR6] Fecke, A., Saw, N. M., Kale, D., Kasarla, S. S., Sickmann, A., & Phapale, P. (2023). Quantitative analytical and computational workflow for large-scale targeted plasma metabolomics. *Metabolites*, *13*, 844. 37512551 10.3390/metabo13070844 PMC10383057

[CR7] Fiehn, O., Robertson, D., Griffin, J., et al. (2007). The metabolomics standards initiative (MSI). *Metabolomics*, *3*, 175–178. 10.1007/s11306-007-0082-2 PMC3772505 24039616

[CR8] Gilar, M., Berthelette, K., & Walter, T. H. (2022). Contribution of ionic interactions to stationary phase selectivity in hydrophilic interaction chromatography. *Journal of Separation Science*, *45*, 3264–3275. 35347885 10.1002/jssc.202200165 PMC9545918

[CR10] Kodra, D., Pousinis, P., Vorkas, P. A., Kademoglou, K., Liapikos, T., Pechlivanis, A., Virgiliou, C., Wilson, I. D., Gika, H., & Theodoridis, G. (2022). Is current practice adhering to Guidelines proposed for metabolite identification in LC-MS untargeted metabolomics? A Meta-analysis of the literature. *Journal of Proteome Research*, *21*, 590–598. 34928621 10.1021/acs.jproteome.1c00841

[CR11] Lioupi, A., Virgiliou, C., Walter, T. H., Smith, K. M., Rainville, P., Wilson, I. D., Theodoridis, G., & Gika, H. G. (2022). Application of a hybrid zwitterionic hydrophilic interaction liquid chromatography column in metabolic profiling studies. *Journal of Chromatography A*, *1672*, 463013. 35436684 10.1016/j.chroma.2022.463013

[CR12] Phapale, P., Palmer, A., Gathungu, R. M., Kale, D., Brügger, B., & Alexandrov, T. (2021). Public LC-Orbitrap tandem mass spectral library for metabolite identification. *Journal of Proteome Research*, *20*(4), 2089–2097. 33529026 10.1021/acs.jproteome.0c00930

[CR13] Schrimpe-Rutledge, A. C., Codreanu, S. G., Sherrod, S. D., & McLean, J. A. (2016). Untargeted metabolomics strategies-challenges and emerging directions. *Journal of the American Society for Mass Spectrometry*, *27*, 1897–1905. 27624161 10.1007/s13361-016-1469-y PMC5110944

[CR14] Schymanski, E. L., Jeon, J., Gulde, R., Fenner, K., Ruff, M., Singer, H. P., & Hollender, J. (2014). Identifying small molecules via high resolution mass spectrometry: Communicating confidence. *Environmental Science & Technology*, *48*, 2097–2098. 24476540 10.1021/es5002105

[CR15] Serafimov, K., & Lämmerhofer, M. (2022). Metabolic profiling workflow for cell extracts by targeted hydrophilic interaction liquid chromatography-tandem mass spectrometry. *Journal of Chromatography A*, *1684*, 463556. 36265203 10.1016/j.chroma.2022.463556

[CR16] Sumner, L. W., Amberg, A., Barrett, D., & Viant, M. (2007). Proposed minimum reporting standards for chemical analysis. *Metabolomics*, *3*, 211–221. 24039616 10.1007/s11306-007-0082-2 PMC3772505

[CR17] Theodoridis, G., Gika, H., Raftery, D., Goodacre, R., Plumb, R. S., & Wilson, I. D. (2023). Ensuring fact-based metabolite identification in Liquid Chromatography-Mass Spectrometry-based metabolomics. *Analytical Chemistry*, *95*, 3909–3916. 36791228 10.1021/acs.analchem.2c05192 PMC9979140

[CR18] Tobolkina, E., González-Ruiz, V., Meister, I., De Figueiredo, M., Guillarme, D., Boccard, J., & Rudaz, S. (2022). Challenges in ESI-MS-based untargeted metabolomics. *Chimia*, *76*, 90. 38069754 10.2533/chimia.2022.90

[CR19] Tsugawa, H., Cajka, T., Kind, T., Ma, Y., Higgins, B., Ikeda, K., Kanazawa, M., VanderGheynst, J., Fiehn, O., & Arita, M. (2015). MS-DIAL: Data-independent MS/MS deconvolution for comprehensive metabolome analysis. *Nature Methods*, *12*(6), 523–526. 25938372 10.1038/nmeth.3393 PMC4449330

[CR20] Walter, T. H., Alden, B. A., Berthelette, K., Field, J. A., Lawrence, N. L., McLaughlin, J., & Patel, A. V. (2022). Characterization of a highly stable zwitterionic hydrophilic interaction chromatography stationary phase based on hybrid organic-inorganic particles. *Journal of Separation Science*, *45*, 1389–1399. 34937126 10.1002/jssc.202100859 PMC9487986

