# Supplementary material for: EMBL-MCF 2.0: an LC-MS/MS method and corresponding library for high-confidence targeted and untargeted metabolomics using low-adsorption HILIC chromatography
Source: Metabolomics. 2024 Oct 13;20(6):114. doi: 10.1007/s11306-024-02176-1 (PMC11471713; doi:10.1007/s11306-024-02176-1)
Supplement: Supplementary file 2 — Supplementary Material 2 [file 11306_2024_2176_MOESM2_ESM.docx]

**Supplementary Information**

**___________________________________________________________**

**EMBL-MCF 2.0: LC-MS/MS library for high-confidence targeted and untargeted metabolomics using low-adsorption HILIC
chromatography**

Svitlana Dekina, Theodore Alexandrov, Bernhard Drotleff

Metabolomics Core Facility, European Molecular Biological Laboratory, Heidelberg 69117, Germany

Structural and Computational Biology Unit, European Molecular Biology Laboratory (EMBL), Heidelberg, Germany

Molecular Medicine Partnership Unit, EMBL and Heidelberg University, Heidelberg, Germany

Bio Studio, BioInnovation Institute, Copenhagen, Denmark

**Contents**

**
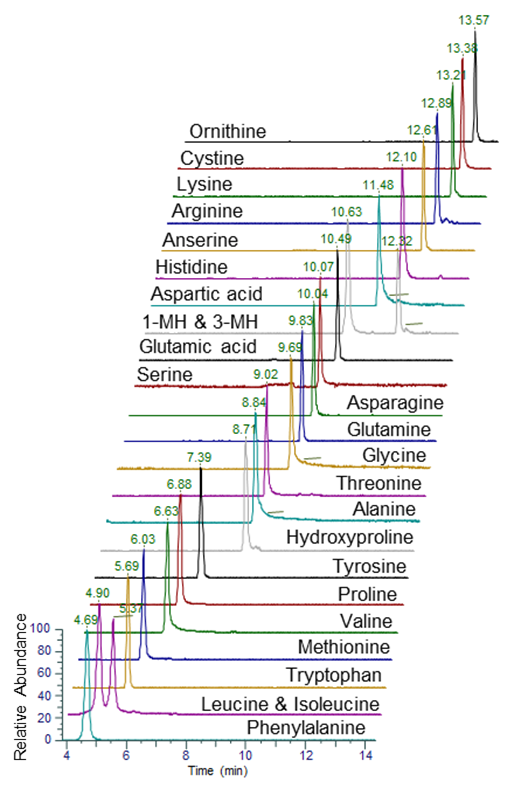
**

**Figure S1.** BEH Z-HILIC chromatograms of 25 amino acids. 1-MH: 1-Methylhistidine, 3-MH: 3-Methylhistidine

**Table S5.** Repeatability of retention times for an Amino Acid Standard mix (Sigma) used as system suitability check (n = 7), max RT variation of 4 s

| **Analyte** | **CV [%]** |
| --- | --- |
| Cystine | 0.23 |
| Lys | 0.24 |
| Arg | 0.27 |
| His | 0.34 |
| Asp | 0.28 |
| Glu | 0.21 |
| Ser | 0.27 |
| Asn | 0.30 |
| Gln | 0.24 |
| Gly | 0.22 |
| Thr | 0.32 |
| Ala | 0.28 |
| Tyr | 0.34 |
| Pro | 0.37 |
| Val | 0.46 |
| Met | 0.42 |
| Trp | 0.46 |
| Leu | 0.61 |
| Ile | 0.75 |
| Phe | 0.71 |

The CV values are based on results acquired for seven individual preparations of mobile phase. Inter-batch variation between columns was not investigated.

**Table S6.** Isomeric compounds covered in the library

| **Compound Name** | **Chemical Formula** | **Polarity** | **RT** |
| --- | --- | --- | --- |
| 2-Deoxyguanosine | C10H13N5O4 | - | 4.67 |
| Adenosine |  |  | 2.35 |
| Adenosine monophosphate | C10H14N5O7P | - | 11.20 |
| Deoxyguanosine monophosphate |  |  | 12.42 |
| Adenosine triphosphate | C10H16N5O13P3 | - | 14.23 |
| Deoxyguanosine triphosphate |  |  | 15.73 |
| Dihydroxyacetone | C3H6O3 | - | 2.49 |
| Glyceraldehyde |  |  | 6.31 |
| Hydroxypropionic acid |  |  | 7.51 |
| Lactic Acid |  |  | 5.86 |
| Alanine | C3H7NO2 | - | 8.82 |
| Beta-Alanine |  |  | 9.66 |
| Sarcosine |  |  | 8.22 |
| Dihydroxyacetone_phosphate | C3H7O6P | - | 12.07 |
| Glyceraldehyde 3-phosphate |  |  | 12.68 |
| Fumaric acid | C4H4O4 | - | 12.95 |
| Maleic acid |  |  | 2.62 |
| 2-Ketobutyric acid | C4H6O3 | - | 2.83 |
| Acetoacetic acid |  |  | 5.01 |
| Methylmalonic acid | C4H6O4 | - | 7.88 |
| Succinic acid |  |  | 11.80 |
| Butyric acid | C4H8O2 | - | 2.80 |
| Isobutyric acid |  |  | 2.48 |
| Homoserine | C4H9NO3 | - | 9.33 |
| Threonine |  |  | 8.95 |
| 5-Aminovaleric acid | C5H11NO2 | - | 10.28 |
| Valine |  |  | 6.71 |
| Citraconic acid | C5H6O4 | - | 2.69 |
| Itaconic acid |  |  | 10.76 |
| Aminolevulinic acid | C5H9NO3 | - | 10.37 |
| Hydroxyproline |  |  | 8.70 |
| N-Acetylalanine |  |  | 6.05 |
| Isoleucine | C6H13NO2 | -  - | 5.49 |
| Leucine |  |  | 5.04 |
| Fructose-1,6-diphosphate | C6H14O12P2 | - | 16.53 |
| Glucose 1,6-diphosphate |  |  | 16.21 |
| cis-Aconitic acid | C6H6O6 | - | 12.89 |
| trans-Aconitic acid |  |  | 14.95 |
| N-Acetylgalactosamine | C8H15NO6 | - | 5.28 |
| N-acetylmannosamine |  |  | 5.70 |
| p-Coumaric acid | C9H8O3 | - | 4.42 |
| Phenylpyruvic acid |  |  | 1.70 |
| Cotinine | C10H12N2O | + | 1.05 |
| Serotonin |  |  | 5.29 |
| Adenosine | C10H13N5O4 | + | 2.44 |
| Deoxyguanosine |  |  | 4.70 |
| Adenosine monophosphate | C10H14N5O7P | + | 10.60 |
| Deoxyguanosine monophosphate dGMP |  |  | 12.51 |
| 2-Aminobutanoic acid | C4H9NO2 | + | 7.74 |
| N,N-Dimethylglycine |  |  | 6.60 |
| Homoserine | C4H9NO3 | + | 9.39 |
| Threonine |  |  | 9.01 |
| 5-Aminovaleric acid | C5H11NO2 | + | 6.14 |
| Betaine |  |  | 5.78 |
| Valine |  |  | 6.62 |
| Aminolevulinic acid | C5H9NO3 | + | 7.66 |
| Hydroxyproline |  |  | 8.72 |
| N-Acetylalanine |  |  | 2.34 |
| Glutamic acid | C5H9NO4 | + | 10.50 |
| O-Acetylserine |  |  | 6.79 |
| Isoleucine | C6H13NO2 | + | 5.37 |
| Leucine |  |  | 4.91 |


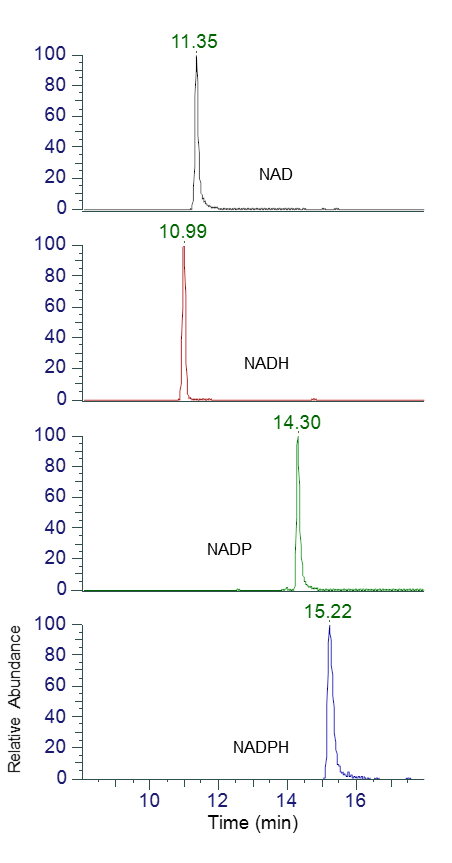


**Figure S2.** BEH Z-HILIC chromatograms of NAD, NADH, NADP and NADPH in ESI-.

**Table S7.** Favorable mode for plasma metabolites detected in both modes for high resolution data (based on precursor ions)

| **Metabolite name** | **Ratio Pos/Neg Area** | **Ratio Pos/Neg Height** | **Precision CV [%]** | | **Favorable mode** |
| --- | --- | --- | --- | --- | --- |
|  |  |  | **pos** | **neg** |  |
| 2-Deoxycytidine | 0.79 | 0.66 | 4.57 | 4.55 | neg |
| Adenosine | 10.33 | 9.28 | 1.82 | 9.92 | pos |
| Adenosine monophosphate | 0.89 | 0.67 | 9.72 | 5.21 | neg |
| Alanine | 3.76 | 2.61 | 5.50 | 5.07 | pos |
| Anserine | 3.97 | 4.53 | 5.16 | 5.06 | pos |
| Arginine | 5.61 | 12.55 | 2.07 | 3.71 | pos |
| Asparagine | 0.87 | 1.49 | 5.15 | 4.64 | pos |
| Aspartic acid | 0.23 | 0.35 | 0.71 | 2.67 | neg |
| Bilirubin | 0.05 | 0.04 | 10.57 | 15.10 | neg |
| Carnitine | 497.21 | 511.79 | 12.18 | 2.85 | pos |
| Citrulline | 1.49 | 1.95 | 2.75 | 6.24 | pos |
| Cytidine | 0.62 | 0.45 | 2.10 | 3.51 | neg |
| Deoxyinosine | 4.66 | 6.00 | 2.68 | 1.46 | pos |
| Glutamic acid | 0.62 | 0.60 | 6.61 | 7.47 | neg |
| Glutamine | 1.15 | 1.30 | 2.53 | 1.23 | pos |
| Glutathione (GSH) | 0.58 | 0.70 | 1.24 | 2.57 | neg |
| Glutathione (GSSG) | 1.59 | 1.59 | 17.01 | 14.59 | pos |
| Glycerol 3-Phosphate | 0.04 | 0.03 | 11.13 | 10.55 | neg |
| Glycine | 0.39 | 0.3 | 10.22 | 4.44 | neg |
| Guanosine | 0.68 | 0.67 | 5.20 | 4.63 | neg |
| Histidine | 0.14 | 0.20 | 5.10 | 7.73 | pos |
| Hydroxyproline | 312.72 | 349.07 | 4.65 | 12.75 | pos |
| Hypoxanthine | 1.35 | 1.3 | 9.06 | 18.50 | neg |
| Inosine | 0.21 | 0.24 | 1.12 | 4.30 | neg |
| Inosinic acid | 0.71 | 0.56 | 4.81 | 2.73 | neg |
| Isoleucine | 3.55 | 4.16 | 11.52 | 12.41 | pos |
| Kynurenic acid | 0.55 | 0.49 | 3.92 | 1.90 | neg |
| Leucine | 3.36 | 3.28 | 5.79 | 4.18 | pos |
| Lysine | 4.61 | 7.78 | 2.71 | 2.33 | pos |
| Methionine | 2.49 | 3.13 | 2.10 | 4.99 | pos |
| N-Acetylglutamine | 0.29 | 0.33 | 3.29 | 3.34 | neg |
| N-Acetylphenylalanine | 0.05 | 0.09 | 2.12 | 7.19 | neg |
| Ornithine | 250.35 | 235.15 | 6.63 | 1.23 | pos |
| Pantothenic acid | 0.91 | 2.38 | 1.79 | 21.52 | pos |
| Phenylalanine | 0.01 | 0.01 | 2.84 | 3.85 | neg |
| Phosphocreatine | 2.95 | 0.66 | 6.29 | 2.00 | neg |
| Phosphoethanolamine | 0.82 | 0.48 | 14.25 | 4.11 | neg |
| Pipecolic acid | 12.87 | 12.16 | 12.21 | 7.05 | pos |
| Proline | 22.19 | 29.38 | 4.52 | 4.16 | pos |
| Pyroglutamic acid | 0.05 | 0.08 | 2.81 | 4.62 | neg |
| S-Sulfocysteine | 0.41 | 0.60 | 4.84 | 5.70 | neg |
| Serine | 0.19 | 0.25 | 4.06 | 2.96 | neg |
| Taurine | 0.97 | 0.94 | 3.55 | 4.59 | pos |
| Taurocholic acid | 0.02 | 0.01 | 1.83 | 2.69 | neg |
| Threonine | 0.83 | 1.49 | 4.79 | 1.12 | pos |
| Thymidine | 0.07 | 0.08 | 4.40 | 1.90 | neg |
| Tryptophan | 1.25 | 1.63 | 10.38 | 3.07 | pos |
| Uric Acid | 0.01 | 0.01 | 1.26 | 2.58 | neg |
| Valine | 2.12 | 2.25 | 6.09 | 2.92 | pos |
| Xanthine | 0.01 | 0.01 | 2.63 | 3.21 | neg |

1. **Parameters for MS-DIAL ver. 4.9.221218**

*#Project*

MS1 Data type Profile

MS2 Data type Profile

Ion mode Negative

Target Metabolomics

Mode ddMSMS

*#Data collection parameters*

Retention time begin 0.5 min

Retention time end 18 min

Mass range begin [*m/z*] 60

Mass range end [*m/z*] 900

MS2 mass range begin [*m/z*] 40

MS2 mass range end [*m/z*] 950

*#Centroid parameters*

MS1 tolerance [*m/z*] 0.01

MS2 tolerance [*m/z*] 0.02

*#Isotope recognition*

Maximum charged number ±2

*#Peak detection parameters*

Smoothing method LinearWeightedMovingAverage

Smoothing level 3

Minimum peak width 10

Minimum peak height 30000

*#Peak spotting parameters*

Mass slice width 0.05

#Deconvolution parameters

Sigma window value 0.5

MS2Dec amplitude cut off 0

Exclude after precursor TRUE

Keep isotope until 0.5

Keep original precursor isotopes FALSE

*#MSP file and MS/MS identification setting*

Retention time tolerance 1 min

Mass tolerance (MS1) [*m/z*] 0.005

Mass tolerance (MS2) [*m/z*] 0.02

Identification score cut off 80

Using retention time for scoring TRUE

Using retention time for filtering TRUE

*#Alignment parameters setting*

Retention time tolerance 0.1 min

MS1 tolerance [*m/z*] 0.005

Retention time factor 0.5

MS1 factor 0.5

Peak count filter 0

N% detected in at least one group 50

Remove feature based on peak height fold-change

TRUE

Sample max / blank average 5

Sample average / blank average 5

Ratio experimental samples : QCs 5:1

1. **Dwell volume and extracolumn volume of the Thermo Vanquish Horizon UHPLC system connected to an Orbitrap Exploris 240**

Gradient delay volume:

- Binary Pump H - Dwell volume: 35 μL
- Split Sampler HT - Dwell volume: 110 μL (including 25 μL loop)
- Fluidic connections: Viper MP35N, 0.100 m x 150 mm - Dwell volume: 4.71 μL

Extracolumn volume:

- Fluidic connections: Viper MP35N, 0.100 m x 250 mm - Dwell volume: 7.85 μL
- HESI needle: 3.14 μL
